# Web-based Machine Learning Model for Predicting Chronic Kidney Disease in Patients with Type 2 Diabetes Mellitus: A Multicenter Study

**DOI:** 10.12688/f1000research.179913.1

**Published:** 2026-05-09

**Authors:** Lily Kresnowati, Suhartono Suhartono, Zahroh Shaluhiyah, Bagoes Widjanarko, Faizul Hasan

**Affiliations:** 1 Doctoral Program of Public Health, Faculty of Public Health, Universitas Diponegoro, Semarang, Central Java, Indonesia; 2 Department of Environmental Health, Diponegoro University School of Public Health, Semarang, Central Java, Indonesia; 3 Department of Health Promotion and Behavioral Science, Faculty of Public Health, Universitas Diponegoro, Semarang, Central Java, Indonesia; 4 Faculty of Nursing, Chulalongkorn University, Bangkok, Bangkok, Thailand

**Keywords:** chronic kidney disease, type 2 diabetes mellitus, machine learning, prediction model, web-based calculator.

## Abstract

**Background:**

Chronic kidney disease (CKD) is a serious complication of type 2 diabetes (T2DM), particularly in low- and middle-income countries with limited access to early diagnosis. Predicting CKD risk using routine clinical data could enable earlier nephroprotective care. This study developed and internally validated a machine learning-based web application to predict incident CKD among T2DM patients in Indonesia’s national health insurance program (Prolanis).

**Methods:**

A machine learning prediction model was conducted using BPJS Prolanis data (2017–2023). Adults (≥18 years) with T2DM and no prior CKD were included. Six algorithms (Logistic Regression, Random Forest, Decision Tree, XGBoost, LightGBM, CatBoost) were trained to predict CKD. Performance was assessed via accuracy, precision, recall, F1 score, and AUC. SHAP was used for interpretability.

**Results:**

Among 7,581 individuals, 864 (11.4%) developed CKD. CatBoost achieved the best performance (AUC = 0.847, accuracy = 0.797, precision = 0.643, recall = 0.525, F1 = 0.578). SHAP identified rapid-acting insulin analogues, amlodipine, furosemide, high blood urea nitrogen, and folic acid as key positive predictors. Advanced age and higher comorbidity burden increased risk, while chronic ischaemic heart disease and dental pulp diseases appeared protective—likely due to healthcare utilization bias. A web-based risk calculator was developed.

**Conclusions:**

The CatBoost-based web app demonstrated strong discriminative ability for predicting incident CKD in T2DM patients using routine claims data. This tool may support risk stratification in primary care settings across Indonesia and similar low-resource environments.

## Introduction

Chronic kidney disease (CKD) constitutes a significant global public health challenge, with particularly concerning trends observed in low- and middle-income nations. ^1, 2^ Characterised by a gradual decline in renal function, CKD significantly elevates the risks of cardiovascular incidents, end-stage renal disease (ESRD), hospitalisation, and early mortality. ^3^ Approximately 10% of persons globally suffer from CKD, with diabetes mellitus and hypertension responsible for more than half of the cases. ^4^ The prevalence of CKD in Indonesia has consistently increased over the last decade, primarily due to the rising incidence of type 2 diabetes mellitus (T2DM), which currently impacts around 10.7 million adults nationwide. ^5, 6^ The economic and societal burdens are significant: ^7, 8^ chronic kidney disease necessitates continuous care, regular monitoring, and, in advanced stages, expensive renal replacement therapy.

Individuals with T2DM are at an elevated risk of developing CKD, referred to as diabetic kidney disease (DKD). Between 20% and 40% of patients with T2DM will develop DKD during their lifetime, establishing diabetes as the predominant cause of End-Stage Renal Disease in numerous countries. ^9– 12^ The etiology of DKD include intricate connections among hyperglycemia, haemodynamic changes, inflammation, and genetic predisposition. ^13, 14^ Hyperglycemia triggers glomerular hyperfiltration, oxidative stress, and the buildup of advanced glycation end-products, ultimately leading to glomerulosclerosis, tubulointerstitial fibrosis, and progressive nephron loss. ^14, 15^ Well-defined risk factors for DKD encompass advanced age, male gender, prolonged diabetes duration, higher haemoglobin A1c levels, hypertension, dyslipidaemia, obesity, and tobacco use. ^9, 16^ Timely diagnosis of T2DM patients at increased CKD risk is essential for the initiation of effective nephroprotective strategies: stringent glycaemic management, blood pressure control through RAAS inhibition, and lifestyle modifications.

Conventional DKD risk prediction techniques predominantly employ logistic regression or Cox proportional hazards, utilising a restricted set of predetermined factors. Despite achieving moderate performance (AUC values generally between 0.70 and 0.80), these models exhibit significant limitations: they presuppose linear relationships between predictors and outcomes, failing to adequately represent the intricate biology of DKD; they frequently necessitate comprehensive data on all predictors, which is often inaccessible in resource-limited environments; and many are developed from specific clinical trial populations or small, single-center cohorts, thereby raising concerns regarding their generalisability. ^17, 18^

Machine learning (ML) provides a robust alternative for predicting clinical risks. Ensemble tree-based methodologies—specifically CatBoost, XGBoost, and LightGBM—exhibit considerable promise owing to their resilience against outliers, capacity to manage heterogeneous data types, and intrinsic mechanisms for addressing missing values, frequently surpassing logistic regression with AUCs ranging from 0.80 to 0.90. ^19, 20^ Nonetheless, significant deficiencies persist: the majority of ML research originates from high-income nations, with scant contributions from Southeast Asia, particularly Indonesia; numerous models depend on laboratory metrics that are not easily accessible in primary care settings; practical clinical application featuring user-friendly web interfaces is restricted; and model interpretability has only recently been tackled via SHAP analysis. ^21^

Indonesia’s national health insurance system (BPJS Kesehatan) encompasses roughly 80% of the populace and administers Prolanis, a chronic disease management initiative that methodically enrols T2DM patients and produces extensive real-world clinical data. Nonetheless, no machine learning-based instrument has been specially designed for the Indonesian T2DM population to forecast incident chronic kidney disease utilising frequently gathered Prolanis data. This study sought to create and internally validate a machine learning predictive model for incident chronic kidney disease in patients with T2DM enrolled in BPJS Indonesia’s national health insurance chronic disease management program (Prolanis), utilising routinely gathered demographic, clinical, and medication data. The study aimed to improve interpretability through SHAP analysis and to offer a practical web-based calculator similar to previously published AI risk assessment tools. ^22^

## Material and methods

### Study design and data source

We predictive ML study utilising a sample dataset from Indonesia’s Badan Penyelenggara Jaminan Sosial (BPJS) Chronic Disease Management Program, referred to as Prolanis. The collection included anonymised patient data gathered from January 1, 2017, to December 31, 2023. The BPJS Prolanis database comprises organised data on patient demographics, clinical diagnoses (classified according to the International Classification of Diseases, 10th Revision [ICD-10]), medication prescriptions (classified using the Anatomical Therapeutic Chemical [ATC] system), laboratory results, and outpatient visit records. The research adhered to the Transparent Reporting of a Multivariable Prediction Model for Individual Prognosis or Diagnosis (TRIPOD) standards.

### Study population

The trial cohort consisted of patients diagnosed with T2DM who engaged in the Prolanis program. Adults (≥18 years) were included if they had a minimum of two documented outpatient visits during the study period. The index date was established as the date of the initial T2DM diagnosis documented within the observation period.

Patients were excluded if they had a previous diagnosis of CKD at the start of their monitoring period. CKD was categorised with ICD-10 codes N18.1–N18.9 (chronic kidney disease stages 1–5, unspecified), alongside Z49 (dialysis care) and Z99.2 (dependency on renal dialysis). Furthermore, patients with absent outcome data or insufficient essential characteristic information were removed from the study.

### Outcome definition

The primary outcome of this study was the onset of incident CKD after the diagnostic date of T2DM. CKD was established by a combination of diagnostic and laboratory criteria to guarantee thorough case identification. A patient was classified as having developed CKD if they met one of the following criteria: (1) a new ICD-10 diagnosis code for CKD (N18.1–N18.9, Z49 for dialysis care, or Z99.2 for renal dialysis dependence) recorded during the follow-up period, or (2) laboratory evidence of renal dysfunction noted in structured clinical documentation, defined as an estimated glomerular filtration rate (eGFR) below 60 mL/min/1.73m ^2^ or the presence of albuminuria. Patients were monitored from the index date until the occurrence of the earliest event: diagnosis of CKD, death, conclusion of accessible data (December 31, 2023), or the last recorded clinical visit, whichever transpired first.

### Data collection and input features

Data at the patient level were retrieved from the BPJS Prolanis database, including demographic details, clinical comorbidities, drug prescriptions, and available laboratory findings. Demographic characteristics encompassed age as a continuous variable (quantified in years), sex (male or female), and overweight or obesity status, defined as a body mass index of 25 kg/m ^2^ or higher when such data were accessible. Comorbidities were detected with ICD-10 codes recorded on the index date or within the year prior; they encompassed hypertension, cardiovascular disease, heart failure, and the aggregate of unique diagnoses as an indicator of overall illness burden. Medication utilisation was assessed using Anatomical Therapeutic Chemical (ATC) codes, with a patient deemed exposed if they had a minimum of one prescription fill for a specific medication within the six months before to or subsequent to the index date. Medications of interest encompassed antidiabetic therapies (notably rapid-acting insulin analogues), aspirin, proton pump inhibitors (PPIs), non-steroidal anti-inflammatory drugs (NSAIDs), amlodipine, furosemide, folic acid, and other antihypertensive or antiplatelet agents. Laboratory data, particularly blood urea nitrogen (BUN) levels, were obtained where accessible. All features, with the exception of age, were binarized to indicate presence or absence, although age was maintained as a continuous variable. Only instances with complete data on essential features and the outcome were included in the final analysis.

### Model development and internal validation

We developed and internally validated six ML algorithms aimed to predict incident CKD: Logistic Regression, Random Forest, Decision Tree, Extreme Gradient Boosting (XGBoost), Light Gradient Boosting Machine (LGBM), and Categorical Boosting (CatBoost). All models were executed utilising the PyCaret open-source machine learning package in Python. The complete dataset was randomly divided into a training set consisting of 80% of the patients and an internal validation (test) set including the remaining 20%, with stratification by outcome status to maintain the proportion of CKD cases in both subsets. Hyperparameters for each algorithm were calibrated using 10-fold cross-validation on the training dataset to enhance model performance and mitigate overfitting. Subsequent to hyperparameter adjustment, each model was trained on the training set and subsequently evaluated on the hold-out test set to determine its predictive efficacy on novel data. The CatBoost model, exhibiting superior overall performance, was chosen as the final model for subsequent interpretation and feature importance analysis.

### Performance metrics

The model’s performance was evaluated using a thorough array of conventional classification measures to facilitate comparison among the six algorithms. Accuracy was determined as the ratio of right predictions (true positives plus true negatives) to total predictions. Precision, defined as the ratio of true positives to the sum of true positives and false positives, was employed to reduce false-positive predictions. Recall, or sensitivity, was computed as the ratio of true positives to the total of true positives and false negatives, indicating the model’s proficiency in accurately identifying actual CKD cases. The F1 score, defined as the harmonic mean of precision and recall, is calculated as 2 times (precision × recall) divided by (precision + recall), offering a balanced assessment of model accuracy that considers both false positives and false negatives. The area under the receiver operating characteristic curve (ROC-AUC) was employed to evaluate the model’s capacity to differentiate between patients who developed CKD and those who did not across various classification thresholds; an AUC of 0.5 signifies random performance, whereas an AUC of 1.0 denotes perfect discrimination.

### Feature importance and explainability

SHapley Additive exPlanations (SHAP) were employed to explain model predictions and assess the contribution of each feature to the ultimate model output. A summary plot was created to illustrate the ten most significant elements. In the SHAP summary graphic, red signifies elevated feature values (augmenting CKD probability), whereas blue denotes diminished feature values (reducing CKD probability). SHAP values were calculated for the CatBoost model, which exhibited the greatest ROC-AUC among all evaluated algorithms.

### Statistical analysis

All statistical analyses were conducted utilising Python (version 3.9) alongside the PyCaret, scikit-learn, and SHAP libraries. Continuous data are expressed as mean ± standard deviation (SD), whereas categorical variables are represented as frequencies and percentages. Baseline parameters were compared between individuals who developed CKD and those who did not, utilising independent t-tests for continuous variables and chi-square testing for categorical data. A two-tailed p-value of less than 0.05 was deemed statistically significant. No correction for multiple comparisons was implemented owing to the exploratory character of the model construction.

### Ethics approval and consent to participate

The study utilised de-identified secondary data obtained from the BPJS Kesehatan Prolanis database. Ethical approval was obtained from the Institutional Review Board of the Faculty of Public Health at Diponegoro University (approval number: 1.EA/KEPK-FKM/2026). The ethics committee waived the requirement for informed consent due to the study’s reliance on retrospective analysis of anonymised secondary data, which entailed no direct interaction with human participants and did not provide researchers with any identifying information at any point. Consequently, no written nor verbal informed permission was acquired, in accordance with the committee’s waiver.

## Results

### Study population and baseline characteristics

A total of 7,581 patients were included in the final analysis. The average age of the cohort was 54.2 years (SD ± 9.0), with a predominance of females (54.1%). The average number of hospital visits per patient was 90.8, and the average number of recorded diagnoses was 30.6. 27.2% of the population exhibited overweight or obesity. Hypertension was the predominant comorbidity at 9.7%, succeeded by cardiovascular disease at 5.1% and heart failure at 0.3%. Antidiabetics were prescribed to 85.6% of patients, aspirin to 51.2%, proton pump inhibitors to 17.4%, and NSAIDs to 1.6%. CKD was identified in 864 patients (11.4%) ( Table 1).

**Table 1.** Demographic and outcome characteristic.

Variables	n (%)
Total patients	7581 (100)
Age, years (mean ± SD)	54.2 ± 9.0
Gender	
Male	3477 (45.9)
Female	4104 (54.1)
Visit numbers, mean	90.8
Overweight/Obesity	2061 (27.2)
Comorbidity	
Hypertension	732 (9.7)
Cardiovascular disease	385 (5.1)
Heart failure	22 (0.3)
Diagnoses count, mean	30.6
Drugs	
Antidiabetics	6493 (85.6)
Aspirin	3879 (51.2)
Proton Pump Inhibitors	1320 (17.4)
NSAIDs	125 (1.6)
Outcome of having CKD	
Yes	864 (11.4)
No	6717 (88.6)

CKD = Chronic Kidney Disease; n = number; NSAIDs = Non Steroid Anti Inflammatory Drugs; SD = standard deviation.

### Model development and internal validation

**Figure 1.**
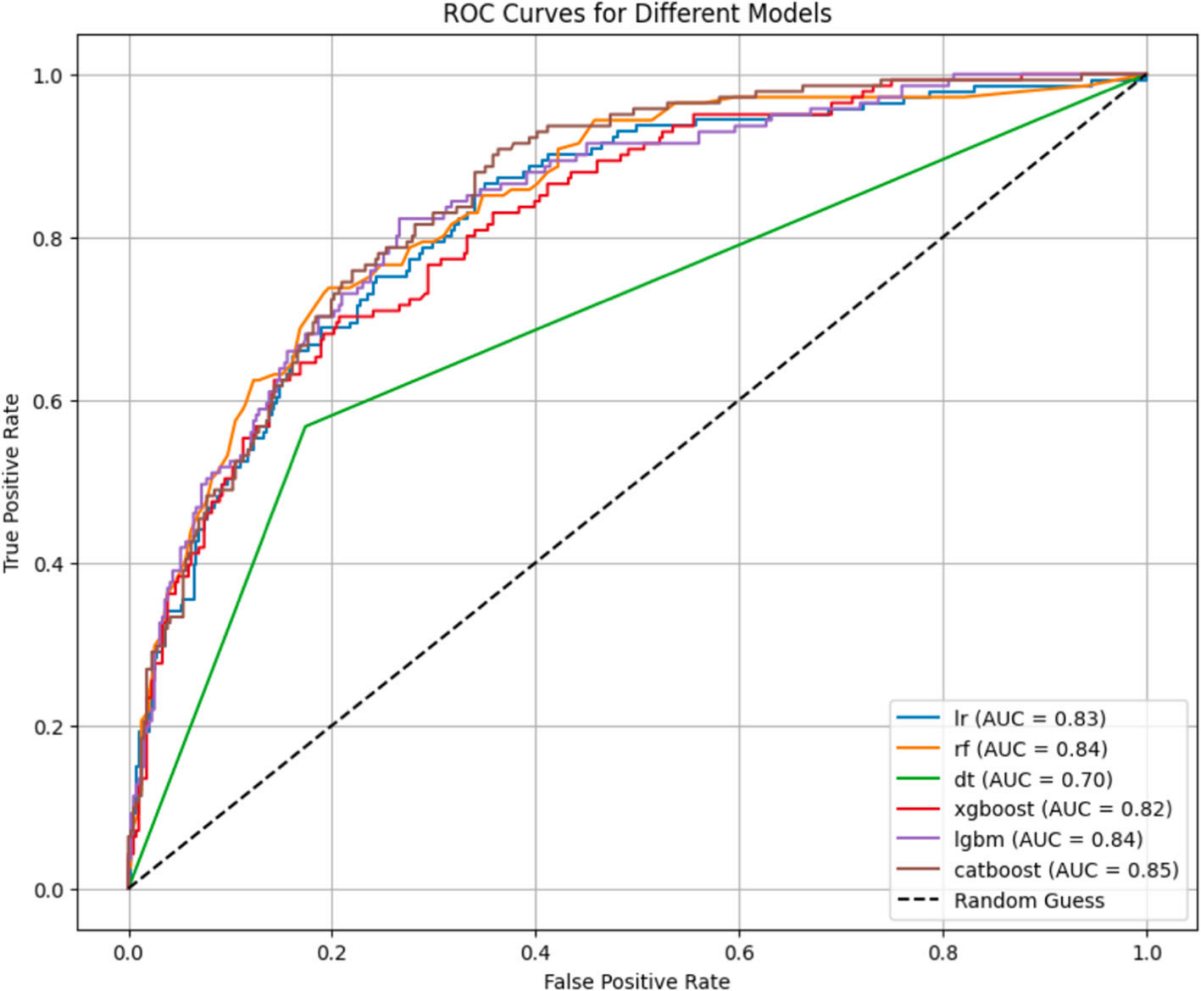
Receiver operating characteristic curve of top 5 model.

Six machine learning algorithms were developed and subjected to internal validation. The CatBoost classifier attained the greatest ROC-AUC of 0.847, succeeded by Random Forest at 0.840, LightGBM at 0.836, Logistic Regression at 0.826, XGBoost at 0.821, and Decision Tree at 0.697. Regarding accuracy, Random Forest exhibited the highest performance at 0.810, whilst both CatBoost and Logistic Regression attained an accuracy of 0.797. CatBoost exhibited a precision of 0.643, a recall of 0.525, and an F1 score of 0.578 ( Table 2). The receiver operating characteristic (ROC) curves for all six models indicated that CatBoost had the highest true-positive rate over the majority of false-positive rate thresholds ( Figure 1).

**Table 2.** Six nearest neighbor algorithms used in machine learning.

Model	Accuracy	Precision	Recall	F1	ROC_AUC
Lr	0.796992	0.651376	0.503546	0.568000	0.826196
Rf	0.810150	0.663934	0.574468	0.615970	0.839900
Dt	0.757519	0.540541	0.567376	0.553633	0.696731
Xgboost	0.798872	0.639344	0.553191	0.593156	0.820736
Lgbm	0.791353	0.622951	0.539007	0.577947	0.835673
Catboost	0.796992	0.643478	0.524823	0.578125	0.847373

Catboost = Categorical Boosting; Dt = Decision Tree; F1 = F1-score; Lgbm = Light Gradient Boosting Machine; Lr = Logistic Regression; Rf = Random Forest; ROC_AUC = Area Under the Receiver Operating Characteristic Curve; Xgboost = Extreme Gradient Boosting.

### Feature importance and SHAP analysis

**Figure 2.**
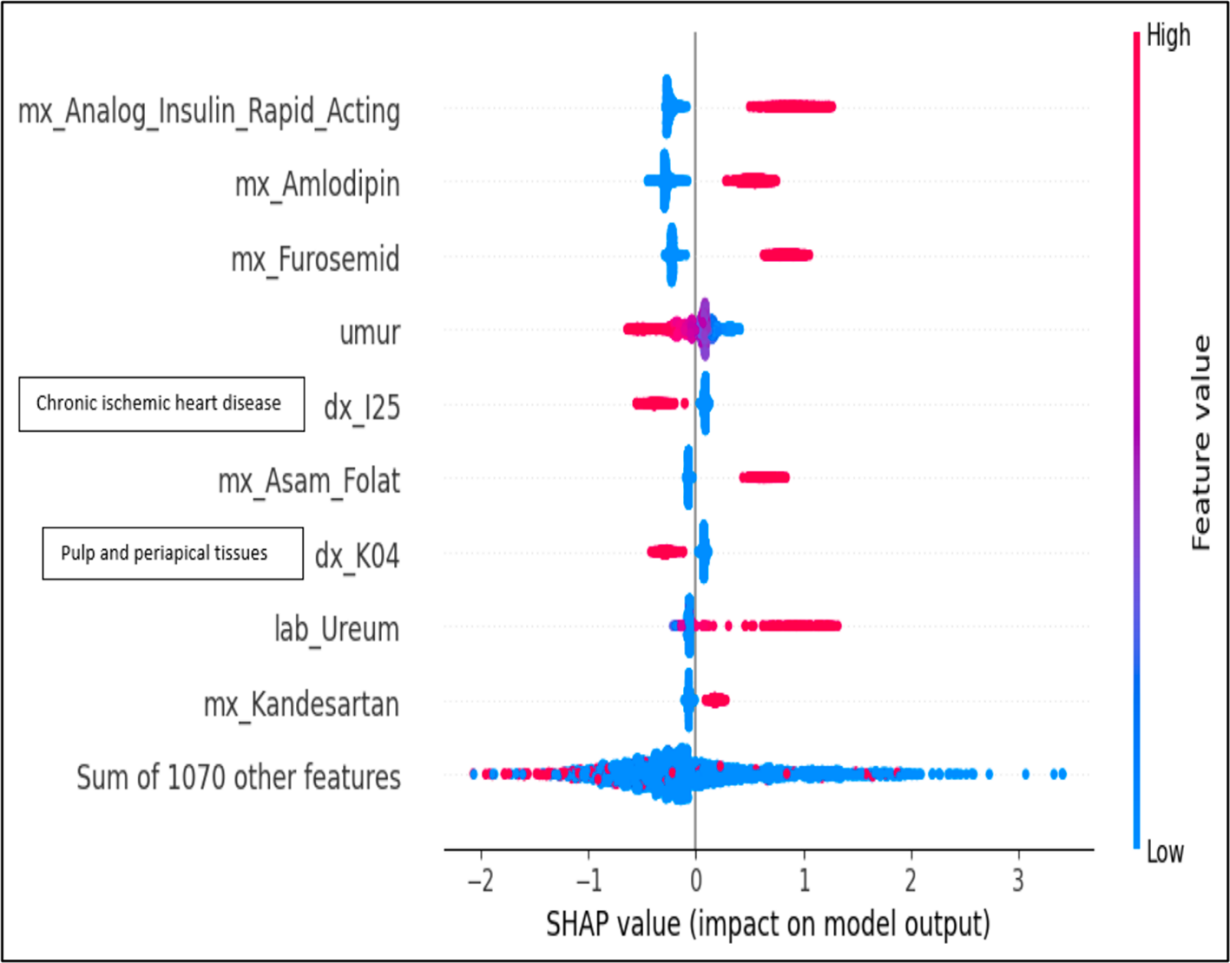
Shapley additive explanations analysis summary plot for top 10 feature important.

SHAP research determined the ten most significant features influencing model predictions. Elevated levels (red) of rapid-acting insulin analogue utilisation were significantly correlated with a heightened likelihood of CKD, whereas diminished levels (blue) were linked to a reduced risk. Likewise, elevated levels of amlodipine, furosemide, folic acid, and BUN augmented the probability of CKD prediction. Conversely, advanced age, chronic ischaemic heart disease, and conditions affecting the pulp and periapical tissues were linked to a diminished predicted chance of CKD ( Figure 2). The protective association of chronic ischaemic heart disease and dental pulp problems may indicate healthcare-seeking behaviours or unaccounted confounding factors in claims-based data.

### Web-based calculator

**Figure 3.**
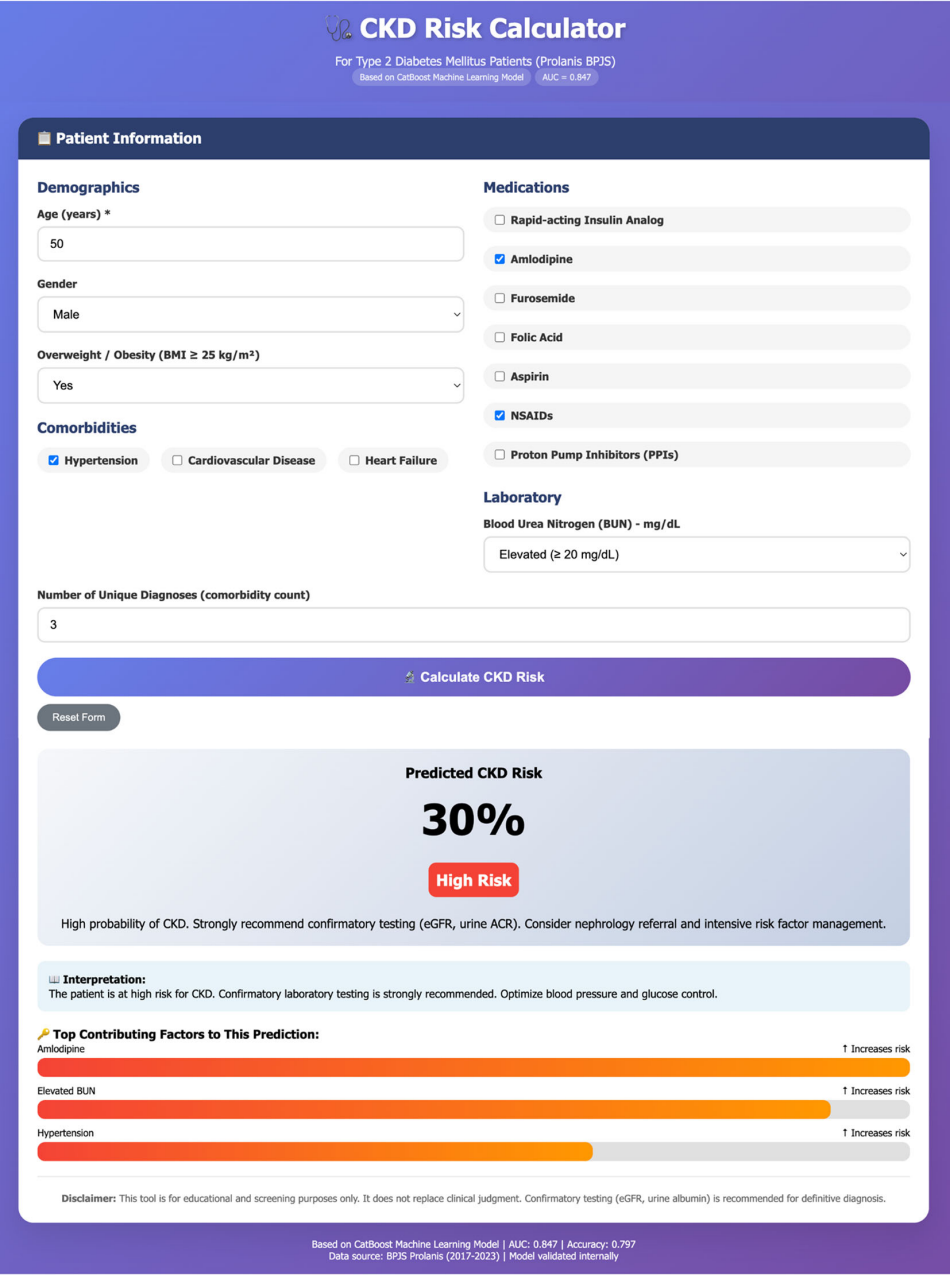
The web-based machine learning calculator system.

A user-friendly web-based risk calculator was built utilising the CatBoost model to enhance clinical application. The calculator enables clinicians to input patient demographics, comorbidities, medications, and BUN values to get a personalised CKD risk assessment. The interface presents the primary contributing elements for each prediction, hence improving model transparency and clinical utility ( Figure 3).

## Discussion

To the best of our knowledge, this study is among the first to develop and validate a web-based machine learning tool for predicting incident CKD in patients with T2DM participating in Prolanis program in Indonesia. In this study of 7,581 patients, we established that the CatBoost classifier attained the superior discriminative performance, evidenced by a ROC-AUC of 0.847, an accuracy of 0.797, and an F1 score of 0.578. The findings indicate that machine learning models, especially gradient boosting algorithms, might function as efficient screening tools able to identify high-risk T2DM patients who could benefit from early nephroprotective therapies.

Our results align with prior research utilising machine learning to predict CKD in diabetic cohorts. A thorough evaluation indicated that machine learning models for predicting diabetic kidney disease generally attain AUC values between 0.80 and 0.90, with gradient boosting techniques frequently surpassing conventional logistic regression. ^19^ A study by Song et al. (2020) utilising the Korean National Health Insurance Service database revealed that XGBoost attained an AUC of 0.84 for predicting CKD progression in T2DM patients, closely corresponding to our CatBoost AUC of 0.847. ^20^ The equivalent efficacy of CatBoost in our analysis underscores the increasing agreement that ensemble tree-based techniques are adept at managing the high-dimensional, heterogeneous clinical data characteristic of real-world electronic health records.

However, our model’s recall (0.525) and F1 score (0.578) were moderate, suggesting that although the model has reasonable overall accuracy, it exhibits limited sensitivity in identifying all real CKD cases. This phenomenon is prevalent in imbalanced datasets where the result (CKD) manifests in merely 11.4% of the population, as observed in our cohort. Similar issues have been documented in other studies; for example, research utilising a Japanese claims database indicated a recall of 0.58 for CKD prediction employing random forests. ^23^ These findings emphasise the necessity for prudence in utilising such models as exclusive decision-making instruments and stress the significance of integrating machine learning predictions with clinical expertise.

Our SHAP analysis discovered numerous significant predictors of incident CKD, many of which are physiologically plausible and align with current clinical knowledge. The utilisation of rapid-acting insulin analogues was one of the most significant predictors of CKD progression, with elevated values (shown in red on the SHAP summary plot) correlating with a heightened chance of CKD occurrence. This finding presumably indicates confounding by indication rather than a direct nephrotoxic effect of insulin. Patients necessitating rapid-acting insulin generally exhibit prolonged diabetes duration, suboptimal glycaemic management, and heightened insulin resistance—each serving as separate risk factors for diabetic kidney damage. The United Kingdom Prospective Diabetes Study (UKPDS) established that intensive glucose-lowering therapy, encompassing insulin, mitigated the progression of microalbuminuria, indicating that insulin administration serves as an indicator of disease severity rather than a causative factor in CKD. ^24, 25^

The finding that the usage of amlodipine and furosemide elevates the probability of CKD can be interpreted as a reflection of the underlying disease burden. Amlodipine, a calcium channel blocker, is frequently used for hypertension, which impacts roughly 9.7% of our cohort and serves as a significant risk factor for the advancement of CKD. Furosemide, a loop diuretic, is frequently used to patients experiencing fluid overload, which may indicate deteriorating renal function. Although certain experimental studies have suggested that calcium channel blockers may expose glomerular capillaries to elevated systemic pressures, findings from major clinical trials, including the African American Study of Kidney Disease and Hypertension (AASK), indicate that CCB-based therapy may provide less renal protection compared with renin–angiotensin system blockade, particularly in patients with hypertensive CKD. ^26^ Consequently, while these agents remain appropriate when clinically indicated, their use—especially as monotherapy—should be accompanied by careful monitoring of renal function, given their comparatively limited renoprotective effects.

In contrast, our SHAP analysis indicated that chronic ischaemic heart disease and conditions affecting the pulp and periapical tissues seemed to confer protective benefits against CKD, a result that necessitates meticulous interpretation. The observed protective effect of chronic ischaemic heart disease may be attributed to healthcare utilisation bias. Patients with diagnosed cardiovascular disease generally experience more frequent clinician visits, enhanced medication adherence (including antihypertensive and antiplatelet medications), and more rigorous control of risk factors than patients without these diagnoses. Research indicates that a high adherence rate to antihypertensive drugs (≥80%) correlates with a 33% decrease in the risk of end-stage renal disease. ^27– 29^ Likewise, the protective influence of pulp and periapical tissue illnesses may indicate superior health-seeking behaviour, as individuals who obtain regular dental care are likely more proactive in managing diabetes. ^30^ Conversely, non-surgical periodontal therapy has demonstrated the capacity to diminish systemic inflammation, perhaps decelerating the progression of CKD, although residual confounding remains a possibility.

The CatBoost model exhibited strong internal validation performance, with a ROC-AUC of 0.847, which is advantageous compared to previously published prediction methods for CKD. A systematic study assessed CKD prediction models and found that most attained AUC values ranging from 0.70 to 0.85, ^17^ positioning our model within the higher spectrum of available methods. Furthermore, our utilisation of standard administrative claims data—rather than specialised laboratory assessments or imaging—augments the model’s scalability and practical relevance in low- and middle-income contexts such as Indonesia, where access to advanced diagnostic testing may be constrained.

Nonetheless, particular aspects of model performance warrant examination. The CatBoost model’s precision (0.643) significantly exceeded its recall (0.525), signifying that while the model predicts CKD, it is accurate about 64% of the time, although it fails to identify nearly half of the actual CKD cases. The compromise between precision and recall is permissible in a screening environment, when the objective is to identify a group of high-risk patients for confirmatory tests rather than to establish definite diagnoses. ^31^ The web-based tool we created enables doctors to modify the classification threshold according to local resources and preferences; for example, a reduced threshold enhances recall (identifying more true cases) while compromising precision (resulting in more false positives necessitating follow-up testing).

This study possesses numerous significant strengths. The utilisation of an extensive, real-world dataset from Indonesia’s national health insurance program (BPJS) ensures significant validity for the Indonesian population and presents a model that can be incorporated into current digital health frameworks. The incorporation of several machine learning methods, accompanied by systematic hyperparameter optimisation and internal validation, adheres to best-practice guidelines for the building of predictive models. Third, employing SHAP analysis improves model interpretability, countering a prevalent critique of “black box” machine learning models in clinical medicine. ^21^ The development of an intuitive web-based calculator enables prospective integration into clinical practice.

However, some limitations must be recognised. The retrospective cohort design includes potential biases associated with secondary data analysis, such as indication bias (as noted with insulin and antihypertensive drugs) and detection bias (patients with more frequent visits are more likely to receive a diagnosis of CKD). Secondly, the dataset was constrained to variables typically gathered in claims data; we could not include significant clinical factors such as smoking status, alcohol intake, physical activity, dietary habits, family history of kidney disease, comprehensive blood pressure readings, haemoglobin A1c levels, or urine albumin-to-creatinine ratios. The lack of these recognised risk indicators may have constrained model performance, especially recall. Third, the utilisation of ICD-10 codes for outcome determination may have led to misclassification bias, given chronic kidney disease is recognised to be under-represented in administrative databases. However, our composite definition, which incorporates laboratory criteria, partially alleviates this problem. The dataset was derived from a singular health insurance program in Indonesia, perhaps constraining its generalisability to other populations with varying genetic backgrounds, healthcare systems, and practice patterns. External validation with separate datasets from other areas or nations is crucial prior to extensive implementation. Fifth, the complete-case analysis, which excluded patients with missing data, may have created selection bias if the missingness was not entirely random. Sixth, we did not conduct external validation utilising a temporally or geographically separate dataset, which is a crucial subsequent step to verify the model’s generalisability and resilience against overfitting.

Future study must emphasise the external validation of the CatBoost model with independent datasets from various healthcare systems in Southeast Asia and beyond. Prospective validation studies would evaluate the model’s real-world efficacy and clinical applicability, encompassing its influence on clinician decision-making and patient outcomes. Subsequent model enhancement could integrate supplementary predictors, including longitudinal changes in eGFR, albuminuria, and haemoglobin A1c, thereby augmenting predictive precision. Ultimately, interventional studies are required to ascertain if machine learning-guided risk classification results in earlier nephrology referrals, enhanced blood pressure and glucose management, and ultimately a decreased incidence of end-stage renal disease in high-risk T2DM patients.

## Conclusions

This study effectively constructed and internally validated a CatBoost-based machine learning model for predicting incident CKD in patients with T2DM, utilising routinely obtained claims data from Indonesia’s BPJS Prolanis program. The model exhibited strong discriminative capability (AUC 0.847) and recognised clinically significant risk factors, such as rapid-acting insulin use, amlodipine, furosemide, and increased BUN levels. The model’s limited recall suggests scope for enhancement, while its elevated precision and interpretability through SHAP analysis underscore its prospective value as a screening instrument for identifying high-risk individuals for focused preventative measures. A web-based calculator was created to enhance clinical application. Future external validation and prospective implementation studies are necessary prior to extensive clinical deployment.

## Data Availability

All datasets supporting this article are accessible via the following link: https://doi.org/10.5281/zenodo.19634415. ^32^ Data are available under the terms of the Creative Commons Attribution 4.0 International. Zenodo: Supplementary data: https://doi.org/10.5281/zenodo.19521819. ^33^ This project contains the following extended data:
• Tripod Checklist.docx. Data are available under the terms of the Creative Commons Attribution 4.0 International.
