# Supplementary material for: Web-based Machine Learning Model for Predicting Chronic Kidney Disease in Patients with Type 2 Diabetes Mellitus: A Multicenter Study
Source: F1000Res. 2026 Jun 10;15:690. Originally published 2026 May 9. [Version 2] doi: 10.12688/f1000research.179913.2 (PMC13305549; doi:10.12688/f1000research.179913.2)
Supplement: Peer review history

# Reviewer report: Reviewer response for version 2

**DOI:** 10.5256/f1000research.202964.r492515

**Published:** 2026-06-25

**Authors:** Cipta Pramana

**Affiliations:** 1 Faculty of Medicine, Universitas Islam Negeri Walisongo Semarang, Semarang, Central Java, Indonesia

"I have carefully reviewed the authors' responses and the revised manuscript (Version 2). The authors have systematically and thoroughly addressed every comment and recommendation from the previous review round.

 Specifically, I appreciate the inclusion of the participant flowchart (Figure 1) which ensures compliance with the TRIPOD statement, the reconstruction of Table 1 to separate the CKD and non-CKD groups with proper p-values, and the correction of the technical typo in Table 2's title.

 Furthermore, the standardization of decimal precision for the AUC values and the meticulous correction of citation formatting errors have resolved all previous technical inconsistencies.

 Crucially, the authors have successfully refined the Abstract, Discussion, and Conclusion sections. The explicit framing of this machine learning model as a preliminary screening tool, alongside the highly practical suggestion of its potential integration into Indonesia's national BPJS P-Care system, substantially strengthens the clinical and practical utility of this study. The future technical recommendations (e.g., SMOTE) also provide excellent direction for subsequent iterations.

 All my previous reservations have been completely resolved, and the manuscript is now methodologically robust, well-presented, and highly relevant. I am very pleased to fully approve this version for indexing."

Is the work clearly and accurately presented and does it cite the current literature?

Yes

If applicable, is the statistical analysis and its interpretation appropriate?

Yes

Are all the source data underlying the results available to ensure full reproducibility?

Yes

Is the study design appropriate and is the work technically sound?

Yes

Are the conclusions drawn adequately supported by the results?

Partly

Are sufficient details of methods and analysis provided to allow replication by others?

Yes

Reviewer Expertise:

"No competing interests were disclosed."

I confirm that I have read this submission and believe that I have an appropriate level of expertise to confirm that it is of an acceptable scientific standard.

# Reviewer report: Reviewer response for version 1

**DOI:** 10.5256/f1000research.198473.r483727

**Published:** 2026-06-04

**Authors:** Ewaldo Eder Carvalho Santana

**Affiliations:** 1 State University of Maranhão, São Luís, Brazil

This study developed and internally validated a machine learning model (CatBoost) to predict incident chronic kidney disease (CKD) among patients with type 2 diabetes mellitus (T2DM) enrolled in Indonesia’s national health insurance chronic disease management program (Prolanis).

 Using a retrospective cohort of 7,581 patients, the authors compared six algorithms and found CatBoost achieved the best discriminative performance (AUC = 0.847). They used SHAP analysis to improve interpretability and built a web‑based risk calculator for clinical use. The manuscript is well structured, follows TRIPOD guidelines, and addresses an important public health gap in low‑ and middle‑income countries. 

 All my answers to the standard review questions were  YES, meaning the study is scientifically sound, the methods are appropriate, the results are clearly presented, the discussion acknowledges limitations, and the conclusions are supported by the data.

Is the work clearly and accurately presented and does it cite the current literature?

Yes

If applicable, is the statistical analysis and its interpretation appropriate?

Yes

Are all the source data underlying the results available to ensure full reproducibility?

Yes

Is the study design appropriate and is the work technically sound?

Yes

Are the conclusions drawn adequately supported by the results?

Yes

Are sufficient details of methods and analysis provided to allow replication by others?

Yes

Reviewer Expertise:

Signal Processing; Machine Learning

I confirm that I have read this submission and believe that I have an appropriate level of expertise to confirm that it is of an acceptable scientific standard.

# Response

**Published:** 2026-06-04

**Authors:** faizul hasan

**Affiliations:** faculty of nursing, Chulalongkorn University, Bangkok, Bangkok, Thailand

We sincerely appreciate your time and insightful evaluation.

 We sincerely appreciate your favorable evaluation and are heartened that you considered the study scientifically robust, the methodologies suitable, and the results well-founded. Your acknowledgement of this work's significance for low- and middle-income countries is particularly important to us.

# Reviewer report: Reviewer response for version 1

**DOI:** 10.5256/f1000research.198473.r483733

**Published:** 2026-05-27

**Authors:** Cipta Pramana

**Affiliations:** 1 Faculty of Medicine, Universitas Islam Negeri Walisongo Semarang, Semarang, Central Java, Indonesia

**Comments for the Authors:**

1. **Introduction (Citation Formatting):** There are several typographical errors regarding text citations in the Introduction section. For instance, in paragraph 2, "3.14 Hyperglycemia triggers..." and "14.15 Well-defined risk factors..." appear to be misformatted reference numbers. Similarly, in paragraph 4, "...from 0.80 to 0.90.19.20" fuses the text with reference numbers 19 and 20. Please thoroughly revise the manuscript to ensure all citations strictly adhere to the journal's formatting guidelines.
2. **Abstract (Methodology):** To improve clarity for a broader audience, please consider adding a brief statement regarding the data-splitting ratio (e.g., 80% training and 20% testing) in the Methods subsection of the Abstract, if the word count permits.
3. **Discussion/Conclusion Alignment:** The authors correctly acknowledged the moderate recall (0.525) due to the imbalanced nature of the dataset. I highly recommend ensuring that the text in the Conclusion explicitly reinforces that this web-based calculator is intended as a preliminary screening tool to guide clinical suspicion rather than a definitive diagnostic instrument. Consistent terminology should also be checked (e.g., matching "ischaemic" vs "ischemic").

**Methodology and Results Comments:**

1. **Methodology (Participant Flowchart):** While the inclusion and exclusion criteria are explicitly defined, the manuscript lacks a participant flow diagram. To adhere strictly to the TRIPOD statement referenced by the authors, please provide a flowchart illustrating how the initial cohort was filtered down to the final sample size of 7,581 patients (e.g., numbers of excluded patients due to prior CKD or missing data).
2. **Table 2 Title Typo:** The title of Table 2 reads *"Six nearest neighbor algorithms used in machine learning."* This is factually incorrect as the models evaluated (CatBoost, XGBoost, Random Forest, etc.) are ensemble tree-based methods and logistic regression, not K-Nearest Neighbor algorithms. Please revise the title to accurately reflect the contents (e.g., *"Performance comparison of the six machine learning models"*).
3. **Results (Table 1 Reconstruction):** In the statistical analysis section, you mentioned comparing baseline parameters between individuals who developed CKD and those who did not using t-tests and chi-square tests. However, Table 1 only displays the aggregate characteristics of the total population. Please reconstruct Table 1 to separate the data into "CKD" and "Non-CKD" groups and include the corresponding p-values to show baseline statistical significance.
4. **Inconsistent AUC Values:** There is a minor inconsistency in how AUC values are reported. For example, CatBoost's AUC is reported as 0.847373 in Table 2, 0.847 in the main text, and 0.85 in Figure 1's legend. Please ensure consistency in decimal precision across the text, tables, and figures (3 decimal places is recommended, e.g., 0.847).

**Discussion and Conclusion Comments for the Authors:**

1. **Discussion (Typographical and Formatting Errors):** There are a few minor formatting issues in the Discussion section. In paragraph 2 (Page 7), an erratic quotation mark appears inside the sentence: "...conventional logistic regression." A study by...". Additionally, on Page 9, a broken reference format is visible: "27- Likewise, the protective influence...". Please meticulously proofread the section to fix these citation alignment errors.
2. **Discussion (Addressing Imbalanced Data):** The authors provided an excellent and honest justification regarding the moderate recall due to the imbalanced nature of the dataset (11.4% CKD prevalence). However, it would strengthen the paper if the authors briefly suggested future technical workarounds in the limitation paragraph—such as utilizing oversampling techniques (e.g., SMOTE) or adjusting algorithmic class weights to improve sensitivity in future iterations.
3. **Conclusions & Practical Implications:** The conclusion is concise and well-balanced. However, given that this study leverages the national BPJS Prolanis dataset, the practical application could be stated more clearly. I suggest the authors add 1–2 sentences addressing how this tool could be realistically integrated into Indonesia's primary healthcare digital ecosystem (e.g., integration into the BPJS P-Care system) to assist general practitioners in risk stratification.

Is the work clearly and accurately presented and does it cite the current literature?

Yes

If applicable, is the statistical analysis and its interpretation appropriate?

Yes

Are all the source data underlying the results available to ensure full reproducibility?

Yes

Is the study design appropriate and is the work technically sound?

Yes

Are the conclusions drawn adequately supported by the results?

Partly

Are sufficient details of methods and analysis provided to allow replication by others?

Yes

Reviewer Expertise:

"No competing interests were disclosed."

I confirm that I have read this submission and believe that I have an appropriate level of expertise to confirm that it is of an acceptable scientific standard, however I have significant reservations, as outlined above.

# Response

**Published:** 2026-06-16

**Authors:** faizul hasan

**Affiliations:** faculty of nursing, Chulalongkorn University, Bangkok, Bangkok, Thailand

Introduction (Citation Formatting): There are several typographical errors regarding text citations in the Introduction section. For instance, in paragraph 2, "3.14 Hyperglycemia triggers..." and "14.15 Well-defined risk factors..." appear to be misformatted reference numbers. Similarly, in paragraph 4, "...from 0.80 to 0.90.19.20" fuses the text with reference numbers 19 and 20. Please thoroughly revise the manuscript to ensure all citations strictly adhere to the journal's formatting guidelines. 

 Response: Thank you for your comment. We have rechecked all references throughout the entire manuscript. As per F1000Research guidelines, references follow a free format but must be consistent across the manuscript. In our study, we have adopted the AMA reference style. 

 Abstract (Methodology): To improve clarity for a broader audience, please consider adding a brief statement regarding the data-splitting ratio (e.g., 80% training and 20% testing) in the Methods subsection of the Abstract, if the word count permits. 

 Response: Thank you for this constructive suggestion. We have revised the Methods subsection of the Abstract to include the data-splitting ratio, as word count permits. The updated sentence now reads: 

 "Six algorithms (Logistic Regression, Random Forest, Decision Tree, XGBoost, LightGBM, CatBoost) were trained on 80% of the data and internally validated on the remaining 20% to predict CKD." 

 Discussion/Conclusion Alignment: The authors correctly acknowledged the moderate recall (0.525) due to the imbalanced nature of the dataset. I highly recommend ensuring that the text in the Conclusion explicitly reinforces that this web-based calculator is intended as a preliminary screening tool to guide clinical suspicion rather than a definitive diagnostic instrument. Consistent terminology should also be checked (e.g., matching "ischaemic" vs "ischemic"). 

 Response: Thank you for this important recommendation. We have made the following revisions: 

 Conclusion section: We have explicitly revised the text to state that the web-based calculator is intended as a preliminary screening tool to guide clinical suspicion, not as a definitive diagnostic instrument. The revised conclusion now reads: 

 “The model's moderate recall indicates potential for enhancement, yet its high precision and interpretability highlight its utility as a preliminary screening tool to inform clinical suspicion and identify high-risk individuals for targeted preventive interventions, rather than serving as an independent diagnostic instrument” 

 Terminology consistency: We have reviewed the entire manuscript and standardized all spelling to "ischaemic" (British English) throughout, ensuring consistency across all sections, including the Discussion, and Conclusion. 

 Methodology and Results Comments: 

 Methodology (Participant Flowchart): While the inclusion and exclusion criteria are explicitly defined, the manuscript lacks a participant flow diagram. To adhere strictly to the TRIPOD statement referenced by the authors, please provide a flowchart illustrating how the initial cohort was filtered down to the final sample size of 7,581 patients (e.g., numbers of excluded patients due to prior CKD or missing data). 

 Response: Thank you for your comment. We agree that a participant flow diagram is essential for TRIPOD compliance. As you noted, we have now provided this flowchart as Figure 1 in the revised manuscript, illustrating the stepwise filtering of the initial cohort down to the final sample of 7,581 patients, including numbers excluded due to prior CKD and missing data. 

 Table 2 Title Typo: The title of Table 2 reads "Six nearest neighbor algorithms used in machine learning." This is factually incorrect as the models evaluated (CatBoost, XGBoost, Random Forest, etc.) are ensemble tree-based methods and logistic regression, not K-Nearest Neighbor algorithms. Please revise the title to accurately reflect the contents (e.g., "Performance comparison of the six machine learning models"). 

 Response: Thank you for catching this error. We have revised the title of Table 2 accordingly. The corrected title now reads: 

 "Performance comparison of the six machine learning models" 

 Results (Table 1 Reconstruction): In the statistical analysis section, you mentioned comparing baseline parameters between individuals who developed CKD and those who did not using t-tests and chi-square tests. However, Table 1 only displays the aggregate characteristics of the total population. Please reconstruct Table 1 to separate the data into "CKD" and "Non-CKD" groups and include the corresponding p-values to show baseline statistical significance. 

 Response: We have reconstructed Table 1 to present baseline characteristics separately for CKD (n=864) and non-CKD (n=6,717) groups, with corresponding p-values from t-tests and chi-square tests. The revised table is included in the manuscript. 

 Inconsistent AUC Values: There is a minor inconsistency in how AUC values are reported. For example, CatBoost's AUC is reported as 0.847373 in Table 2, 0.847 in the main text, and 0.85 in Figure 1's legend. Please ensure consistency in decimal precision across the text, tables, and figures (3 decimal places is recommended, e.g., 0.847). 

 Response: Thank you for noting this inconsistency. We have standardized all AUC values to 3 decimal places (e.g., 0.847) throughout the manuscript, including the main text, Table 2, and Figure 1 legend. 

 Discussion and Conclusion Comments for the Authors: 

 Discussion (Typographical and Formatting Errors): There are a few minor formatting issues in the Discussion section. In paragraph 2 (Page 7), an erratic quotation mark appears inside the sentence: "...conventional logistic regression." A study by...". Additionally, on Page 9, a broken reference format is visible: "27- Likewise, the protective influence...". Please meticulously proofread the section to fix these citation alignment errors. 

 Response: Thank you for this note. We have corrected both the erratic quotation mark on Page 7 and the broken reference format on Page 9. The entire Discussion section has been carefully proofread and revised to ensure all citation formatting is accurate and aligned. 

 Discussion (Addressing Imbalanced Data): The authors provided an excellent and honest justification regarding the moderate recall due to the imbalanced nature of the dataset (11.4% CKD prevalence). However, it would strengthen the paper if the authors briefly suggested future technical workarounds in the limitation paragraph—such as utilizing oversampling techniques (e.g., SMOTE) or adjusting algorithmic class weights to improve sensitivity in future iterations. 

 Response: We thank the reviewer for the positive feedback on our data justification. We agree that highlighting technical workarounds for future research enhances the manuscript. Accordingly, we have revised the limitations paragraph in the Discussion section to explicitly mention the potential use of oversampling techniques (such as SMOTE) and the adjustment of algorithmic class weights to optimize sensitivity/recall in future iterations. 

 "... The moderate recall noted in this study indicates the intrinsically uneven character of the dataset, considering the 11.4% prevalence of CKD. To address this in future iterations, technical solutions such as employing sophisticated oversampling methods (e.g., Synthetic Minority Over-sampling Technique, SMOTE) or systematically modifying algorithmic class weights could be investigated to enhance model sensitivity." 

 Conclusions & Practical Implications: The conclusion is concise and well-balanced. However, given that this study leverages the national BPJS Prolanis dataset, the practical application could be stated more clearly. I suggest the authors add 1–2 sentences addressing how this tool could be realistically integrated into Indonesia's primary healthcare digital ecosystem (e.g., integration into the BPJS P-Care system) to assist general practitioners in risk stratification. 

 Response: We thank the reviewer for this excellent and practical recommendation. We agree that contextualizing the deployment pathway within Indonesia's existing digital health infrastructure significantly strengthens the clinical utility of the study. Accordingly, we have updated the final paragraph of the manuscript to explicitly propose the integration of our CatBoost-based predictive tool into the national BPJS P-Care platform to aid primary care physicians in automated risk stratification. 

 “This web-based calculator is ideal for Indonesia's primary care digital environment as a preliminary screening tool. The CatBoost algorithm embedded directly into the national BPJS P-Care system would allow general practitioners to perform real-time risk stratification during routine visits and provide timely nephroprotective interventions for high-risk type 2 diabetes patients in the Prolanis program.” 

 Is the work clearly and accurately presented and does it cite the current literature? 

 Yes 

 Is the study design appropriate and is the work technically sound? 

 Yes 

 Are sufficient details of methods and analysis provided to allow replication by others? 

 Yes 

 If applicable, is the statistical analysis and its interpretation appropriate? 

 Yes 

 Are all the source data underlying the results available to ensure full reproducibility? 

 Yes 

 Are the conclusions drawn adequately supported by the results? 

 Partly 

 Response: Thank you for your acknowledgement and consideration.
